# Emulsification Properties of Garlic Aqueous Extract: Effect of Heat Treatment and pH Modification

**DOI:** 10.3390/foods12203721

**Published:** 2023-10-10

**Authors:** Ángela Bravo-Núñez, Matt Golding, Manuel Gómez, Lara Matia-Merino

**Affiliations:** 1 Food Technology Area, College of Agricultural Engineering, University of Valladolid, 34071 Palencia, Spain; angela.bravo@uva.es (Á.B.-N.); mgpallares@uva.es (M.G.); 2 School of Food and Advanced Technology, Massey University, Palmerston North 11222, New Zealand; m.golding@massey.ac.nz

**Keywords:** emulsions, garlic extract, surface activity, saponins, heat-treatment, pH

## Abstract

Despite the broad research available in the literature dealing with garlic health benefits, little information is found regarding the functional properties of garlic components. The aim of this study was to evaluate the emulsification properties of garlic water-soluble compounds (GWSC), encompassing proteins, saponins, and carbohydrates, after heat treatment (10 min at 95 °C) or pH adjustments (2.5, 3.5, and 7.8). After the various treatments, the extracts were used as such or filtrated (0.45 µm), and 10% soybean oil-in-water emulsions were prepared using low (0.48%) or high (6.55% wt/wt) extract concentrations. Results showed that whereas at low GWSC concentrations, both heating and acidifying resulted in the formation of bigger oil droplet sizes (i.e., from d_32_ = 0.36 µm using unmodified extract to d_32_ = 7–22 µm at pH 2.5 with or without extract filtration), the effects were opposite at the highest GWSC concentration. In the latter, heat treatment clearly reduced the droplet size as observed from the micrographs as well as the degree of creaming, though the occurrence of depletion and/or bridging flocculation was still strong. The acidification of the extract at this high GWSC concentration significantly reduced the droplet size, as observed from the micrographs; however, a strong flocculation was observed. Removal of protein aggregates, and possibly also saponin micelles, from the extract resulted in an obvious increase in emulsion droplet size. This research brings valuable insights on this study and utilisation of novel natural food emulsifiers from plant sources.

## 1. Introduction

Garlic (*Allium sativum*), a bulbous plant well-known for its health benefits, has been widely used for the treatment of diseases since ancient times [1]. Despite the broad research available regarding its health benefits, little information is available on the functional properties of garlic components. In fact, to our knowledge, only our previous study [2]—dealing with the emulsification properties of garlic extract—together with another study focusing on the emulsification potential of a garlic residual mother liquor (RML), a by-product from the distillation of garlic oil [3], have focused on this matter. 

In our previous study, it was proven that garlic aqueous extracts do include surface-active compounds that, even at low concentrations (0.48% wt/wt), can form relatively stable 10% oil-in-water (O/W) emulsions comprising sub-micron-sized droplets (d_32_ = 0.36 µm) [2]. However, droplet aggregation and accelerated creaming can be observed at higher extract concentrations (6.55% wt/wt) due to a combination of effects involving increasing emulsion droplet size formation (d_32_ = 6.55 µm), depletion flocculation, and bridging phenomena [2]. The underpinning reason for these phenomena was related to the coexistence of compounds of different surface-active natures (i.e., a combination of saponins and proteins/peptides) and the interactions between them and with fructans present in garlic [2].

In the study of An et al. [3], the authors precipitated with ethanol and dried the GWSC, using this powder as the emulsifier agent at various concentrations (0.5–2% *w*/*v*) to stabilise 1% O/W emulsions, obtaining very small droplet sizes. These authors also pointed out that GWSCs that were not precipitated with ethanol presented a much more limited emulsifying potential. These authors focused on the influence that garlic proteins and polysaccharides had on the emulsifying capacity and evaluated the effect of acid hydrolysis of polysaccharides and enzymatic deproteinization on the emulsifying potential of ethanol-precipitated GWSC.

We believe that further understanding of the behaviour of garlic water-soluble extracts as emulsifiers is of special interest, as if these extracts are to be used by the food industry, exposure to high temperatures is often encountered during sterilization, as is the challenge of emulsifying oil in acidic environments, typical of a range of processed food products. To the best of our knowledge, the effect of environmental factors such as heat or a wide range of pHs on the performance of garlic water-soluble extracts as emulsifiers has not been extensively investigated (as previously mentioned, only An et al. [3] addressed environmental factors on dry garlic extracts). 

It is known that the effectiveness of an emulsifier can be affected by the media conditions—such as pH; ionic strength; heating; and freezing [4,5,6]. In fact, there is evidence of the interfacial behaviour of *Quillaja* bark saponins being affected by pH, with the saponins becoming more surface active (reflected in a surface tension decrease) when pH was lowered from pH 7.0 to pH 3.0 [7]. However, the thickness of the foam films formed with these solutions was lower at pH 3.0 compared to pH 7.0. Regarding garlic proteins, only the study by An et al. [3] is available, though there are many reports about the influence of environmental factors on the performance of other proteins, such as milk proteins [8] or vegetal proteins (pea, soy, lentil, and canola) [9], among others. In the case of fructans, to the best of our knowledge, there are only two studies that deal with their interfacial behaviour [10], and no information about the effect of environmental stresses is available. Nevertheless, there are studies about the effect of acid hydrolysis [11] or temperature [12] on fructan structure.

The ionic nature of the steroidal saponins detected in garlic [2], as well as the presence of charged proteins and peptides, make it likely that pH and temperature will have some impact on the emulsification properties. In addition, one of the causes of emulsion instability we have hypothesised is the possibility that proteins may be linked to or complexed with the fructans and/or saponins in the native state of garlic. Therefore, the modification of various garlic aqueous extracts in order to try to modify/denature, and remove larger aggregates involving the biopolymers present in the extract (proteins and polysaccharides)—visible in the transmission electron microscopy images of our previous research [2] and likely to be involved in droplet aggregation—seems highly interesting to be able to control emulsion stability as well as shed light on the relative contributions of the surface-active species in garlic.

Given this background, the research objective of the present work was to study the effect of pH and heat on the emulsification properties of garlic aqueous extracts—by the characterization of 10% oil-in-water emulsions made with garlic extracts (0.48 and 6.55% wt/wt), previously modified by heat or pH treatments and filtrated through a 0.45 µm pore membrane, to remove the visible aggregates (likely formed by aggregated proteins and polysaccharides) [2]. Microstructural analysis—droplet size by static light scattering and light microscopy—along with phase separation measurements of the emulsions under storage were also investigated.

## 2. Materials and Methods

### 2.1. Materials

Fresh peeled garlic was purchased from Marlborough Garlic LTD (Marlborough, New Zealand) and frozen upon arrival (−18 °C). Garlic was then defrosted overnight at 4 °C before experiments. The total solids content of the garlic bulbs was 32.7 ± 0.75 (wt/wt%). Soya bean oil (containing antioxidant E-319) was purchased from Gilmours (Palmerston North, New Zealand). Sodium Hydroxide (NaOH) and hydrochloric acid (HCl) were purchased from Sigma-Aldrich (Saint Louis, MO, USA).

### 2.2. Methods

#### 2.2.1. Extraction of Garlic Water-Soluble Compounds (GWSC)

The aqueous extract was prepared as reported in our previous study [2]. Firstly, defrosted garlic bulbs were blended with reverse osmosis (RO) water (1.78 or 28.44 g garlic/100 g RO water) with a Nutri Ninja^®^ slim blender (SharkNinja Operating LLC, Needham, MA, USA) at room temperature (1 min, 20 °C). The blended garlic + water mixtures were heated up to 50 °C in a water bath for 2 h under continuous stirring to favour the extraction of the garlic water-soluble compounds (GWSC). Then, the mixtures were centrifuged for 30 min at 14,000× *g* and at 20 °C (Thermo Scientific Sorval RC 6+, Waltham, MA, USA). The supernatants were then filtered with Whatman^®^ qualitative filter paper, Grade 1, under vacuum conditions at room temperature. The concentration of GWSC in the extracts (0.48 and 6.55% wt/wt) was calculated as in Bravo-Núñez et al. [2]. Garlic extracts were subsequently used for the following experiments: Extracts were prepared and measured, at least in duplicate. 

#### 2.2.2. Modification of Garlic Aqueous Extract Compounds

The extracts obtained as described before were modified either by a heat treatment (10 min at 95 °C in a water bath with temperature control under continuous stirring) or pH adjustment (pHs of 2.5, 3.5, and 7.8). using a solution of 0.1–1 M HCl (or 0.1–1 M NaOH). These extracts were then filtrated through 0.45 µm pore filters (Millipore Corp., Bedford, MA, USA) in order to remove denatured biopolymers/big aggregates, and some were used as controls (no filtration was carried out). The modification of garlic extracts was performed in duplicate. 

#### 2.2.3. ζ-Potential and Isoelectric Point of Garlic Aqueous Extract Compounds

The zeta potential (ζ) of unmodified GWSC (concentration of soluble compounds of 0.28% wt/wt) was measured using a Zetasizer Nano ZS (ZEN 169 3600) instrument (Malvern Instruments Ltd., Malvern, Worcestershire, UK) using a disposable, folded capillary cell (DTS 1060) at 25 °C. Z-potential was also measured at different pHs to determine the isoelectric point of the extract. Solutions of 0.1–1 M HCl (or 0.1–1 M NaOH) were used to adjust the pH before measurements. Measurements were performed in duplicate in two different batches. 

#### 2.2.4. Emulsion Preparation 

10% (wt/wt) oil-in-water emulsions were prepared by emulsifying soy bean oil and an aqueous phase containing the previously described modified GWSC, following the procedures earlier described in Bravo-Núñez et al. [2]. Soy bean oil and the modified garlic aqueous extract mixture were heated up to 50 °C and subsequently pre-homogenized using a Silverson mixer (Silverson Machines Ltd., Chesham, UK). The coarse emulsions were then homogenized with three passes through a two-stage high-pressure homogenizer (APV 2000; Copenhagen, Denmark) with operating pressures of 25 MPa and 5 MPa for the first and second stage valves. Sodium azide solution (0.02% *v*/*v*, 5 M) was added to the emulsions as an antimicrobial agent. Samples were stored at 4 °C. All emulsions were prepared and analysed in duplicate.

#### 2.2.5. Droplet Size Measurement

The droplet size distribution of the emulsions was determined by the laser light scattering technique using a Malvern Mastersizer MS 2000 (Malvern Instruments Ltd., Worcestershire, UK). Deionized water was used as a dispersant, and the relative refractive index (N) of the emulsion was 1.105, i.e., the ratio of the refractive index of soy oil (1.470) to that of the aqueous medium (1.33). The droplet size distribution of the emulsion droplets was analysed on the same day of preparation and on the following days (1st, 2nd, 3rd, and 7th days) for emulsion stability quantification over time. Emulsions were kept at 4 °C between measurements, and in order to obtain representative results, the emulsions were always manually homogenised before sampling. The volume-mean droplet diameter (*d*_43_) and surface-mean droplet diameter (*d*_32_) were reported. Emulsions were prepared and measured in duplicate.

#### 2.2.6. Light Microscopy 

A light microscope, the Olympus BX53, was used to visualise the microstructure of the emulsions. An aliquot portion of the emulsion sample was placed on a microscope slide. A cover slip was placed on top of the well, ensuring that no air bubbles were trapped inside. The images were captured at ×10, ×40, and ×100 magnifications. Emulsions were prepared and measured in duplicate (measurements were performed the same day of preparation and on subsequent days, the 1st, 2nd, 3rd, and 7th days). A total of ten pictures were taken per sample, and representative images are presented here. 

#### 2.2.7. Statistical Analysis

The data were analysed using a one-way analysis of variance (simple ANOVA). When significant (*p* < 0.05) differences were found, the Fisher’s least significant differences (LSD) test was used to determine the differences among means. Statistical analyses were completed using Statgraphics Centurion XVI software (StatPoint Technologies Inc., Warrenton, VA, USA, EE.UU.). 

## 3. Results and Discussion

### 3.1. Effect of Heat Treatment on the Emulsification Properties of Garlic Aqueous Extract Compounds

#### 3.1.1. Particle Size Distribution

**Figure 1:**
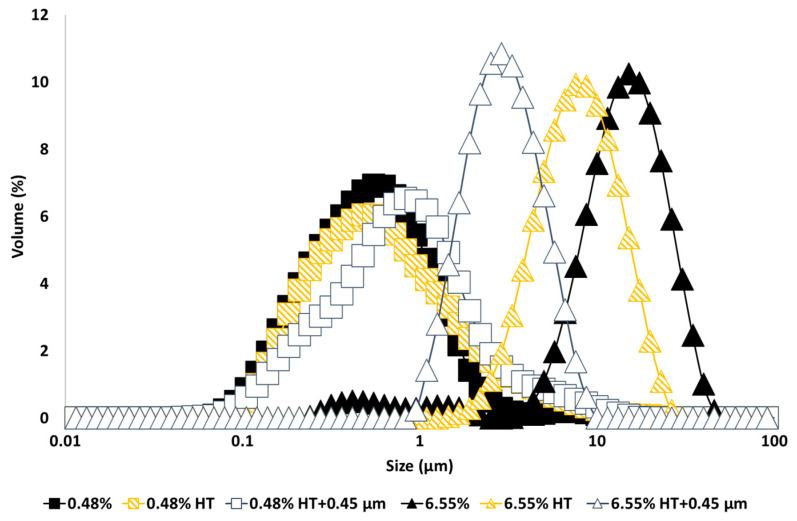
Particle size distribution of 10% oil-in-water emulsions made with unmodified (0.48% and 6.55%), heat-treated (HT), and heat-treated + filtrated (HT + 0.45 μm) GWSC extracts.

The droplet size distribution of emulsions made with untreated and heat-treated GWSC extracts (Figure 1) shows that a smaller droplet size distribution was found for emulsions made at the lowest GWSC concentration, as opposed to using the highest GWSC concentration (6.55%), following the same trend as previously reported [2]. The surface properties of water-soluble garlic extracts come from the various species previously identified that are likely to compete and/or interact at the interface (mainly proteins/peptides, saponins, and fructans). The concentrations of these compounds in the untreated extracts are shown in Table 1. Recent work has demonstrated that pure native Agave fructans do not show any emulsifying capacity or surface activity (Ignot-Gutiérrez et al. [13]); therefore, it is unlikely that these compounds are responsible for any emulsification effect unless they are associated with proteins and/or saponins.

Interestingly, heat treatment of the extract and removal of the larger aggregates by filtration had the opposite effect on the emulsification properties depending upon concentration; whereas at the lowest GWSC concentration (0.48% wt/wt), the emulsion droplet size slightly increased when using heat-treated + filtrated extract (d_32_ = 0.48 µm) versus unmodified extract (d_32_ = 0.36 µm), at the highest GWSC concentration (6.55% wt/wt), there was a reduction in the droplet size when the emulsions were made with heat-treated (d_43_ = 8.18 µm; d_32_ = 6.32 µm) and heat-treated + filtrated extracts (d_43_ = 3.03 µm; d_32_ = 2.46 µm) versus the unmodified one (d_43_ = 12.46 µm; d_32_ = 5.42 µm). The removal of the aggregates of the untreated GWSC also had a positive effect on droplet sizes (d_43_ = 9.24 µm; d_32_ = 7.21 µm) although less pronounced than the removal of the aggregates after heat treatment of the GWSC. Clearly, the removal of the aggregated heat-sensitive compounds and previously existing aggregates (bigger than 0.45 µm) had a positive effect on the emulsifying properties of the GWSC extract at high concentrations. This supports the hypothesis that the interface may be dominated by heat-sensitive biopolymers (likely proteins linked to or not to fructans) when using the unmodified extracts at these concentrations. The denaturation of globular proteins and the removal of these aggregates (linked or not to polysaccharides) by heat and filtration may change the ratio of polymers to saponins, rendering an overall positive effect on the droplet size achieved by allowing more preferential adsorption of the saponin fraction. On the other hand, at the lowest concentration (0.48% wt/wt), the removal of the heat-sensitive biopolymers results in a mild negative effect as slightly bigger droplets are formed, though overall, the droplet sizes are still much smaller than at high GWSC with or without treatment, showing that the interface is still potentially dominated by saponins over polymers, as it has been shown earlier when these species are competing at low protein concentrations [14,15].

**Figure 2:**
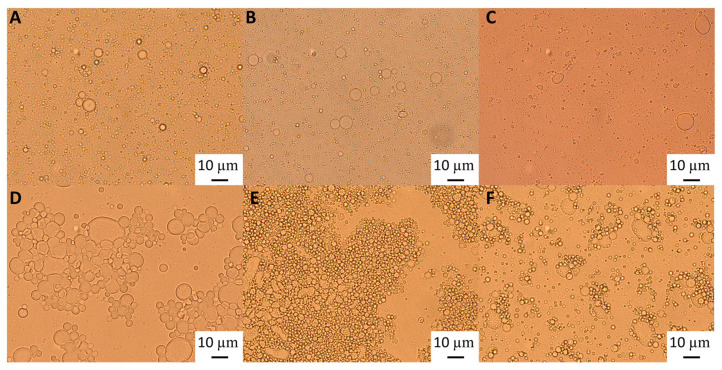
Light microscopy of 10% oil-in-water emulsions made with 0.48% wt/wt GWSC: (**A**) unmodified, (**B**) heat-treated (HT), and (**C**) heat-treated + filtrated (HT + 0.45 μm), and emulsions made with 6.55% wt/wt GWSC: (**D**) unmodified, (**E**) heat-treated (HT), and (**F**) heat-treated + filtrated (HT + 0.45 μm).

Light microscopy images (Figure 2) show that although after heat treatment the droplet size of the emulsions formed with the highest GWSC was reduced (Figure 2E), there are still strong flocculation phenomena being observed—attributed to depletion and bridging mechanisms—as reported in our previous research [2].The depletion phenomenon occurs when non-adsorbing biopolymers or surfactant micelles in the aqueous phase of an emulsion cause an increase in the attractive forces between the droplets due to an osmotic effect associated with the exclusion of the non-adsorbing species from the narrow region between approaching droplets [16,17]. As previously observed with untreated extracts [2], at very low concentrations of free polymers (0.48% wt/wt), the entropy loss linked to particle aggregation outweighs the depletion effect, and the system remains stable even after heat treatment and filtration (Figure 2B,C). 

**Figure 3:**
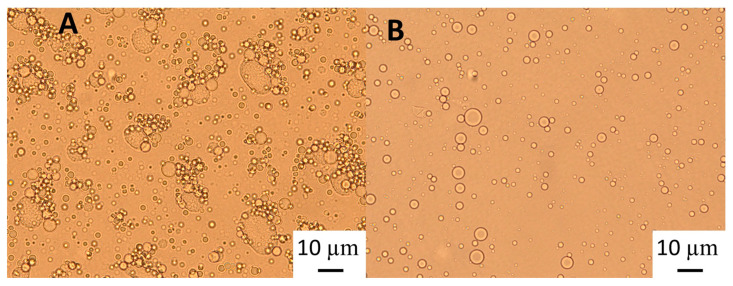
Light microscopy of 10% oil-in-water emulsions of an emulsion stabilised with 6.55% wt/wt GWSC: (**A**) heat-treated + filtrated (HT + 0.45 μm), and (**B**) heat-treated + filtrated (HT + 0.45 μm) mixed with 2% SDS solution (1:1 ratio).

The polymers causing the aggregation phenomenon at high GWSC concentrations (6.55% wt/wt) can be partially removed through filtration, considerably decreasing droplet aggregation (Figure 2F). Nevertheless, some flocs could still be observed after removal of the denatured polymers through filtration, which suggests that not only depletion flocculation phenomena but also bridging flocculation phenomena are taking place in these emulsions (Figure 2E). Figure 3 shows that when mixing the emulsion made with the heat-treated + filtrated extract at the highest concentration (6.55% wt/wt) with SDS, all the flocs disappeared. SDS is used to displace any protein/polysaccharide adsorbed at the interface, confirming the occurrence of bridging flocculation phenomena in both Figure 2E,F as observed in emulsions where proteins and polysaccharides are involved in coating the droplets [18].

This is interesting to note, as it shows that although heat treatment and filtration of the extracts can be applied to reduce droplet size distribution, there are still some surface active compounds remaining in the extracts after the filtration, responsible for the bridging and depletion flocculation phenomena. These compounds could be heat-stable proteins, fructans, and saponin micelles—which are also proven to cause depletion flocculation effects; as high levels of non-adsorbed surfactant micelles are known to increase the osmotic pressure acting on oil droplets, thereby increasing their tendency to flocculate [6]—or aggregates smaller than 0.45 µm. As previously stated, 0.45 µm was chosen as a cutoff to remove big aggregates involving the biopolymers present in the extract and, as demonstrated in this research, partially involved in droplet aggregation. 

According to the literature, above a critical concentration, saponins are capable of forming micellar structures. This concentration varies among different studies—0.08–0.1%wt [19], 0.013 and 0.198 g/L [20], 0.1%wt [21], 02–0.7 g/L [22], 0.5–0.7 g/L [23], or 0.1–0.8 g/L [24]—being the differences explained by the purity and variation in the saponin composition as well as the saponin source. The reported size of these micelles is relatively small (i.e., Samal et al. [21] reported saponin micelle sizes in the range of 10–11.5 nm, while Tippel et al. [25] reported saponin micelles of 6.5 nm). Because no research is available on the physicochemical properties of garlic saponins, the critical micelle concentration (CMC) of these saponins currently remains unknown. Nevertheless, knowing the saponin concentration of our extracts—0.06%/0.6 g/L (0.48% wt/wt GWSC) and 0.9%/9 g/L (6.55% wt/wt GWSC)—leads us to suspect that saponin micelles are likely present at the highest GWSC concentration after filtration; and therefore they could be partially responsible for the depletion flocculation effect observed after both heat treatment and filtration. Droplet bridging at relatively low saponin concentrations (0.1–1% *w*/*v*) has been previously reported in the literature in emulsions co-stabilised with proteins and saponins [26]. Having said that, some of these micelle agglomerates initially present in the GWSC may equally be removed with filtration, as Samal et al. [21] reported saponin micelle sizes in the range of 10–11.5 nm, which can also form agglomerates of sizes ranging from 132–235 nm, 390–990 nm, and 5155–8520 nm, regardless of the saponin concentration in the solution (up to 30 g/L). 

#### 3.1.2. Emulsion Stability over Time 

**Figure 4:**
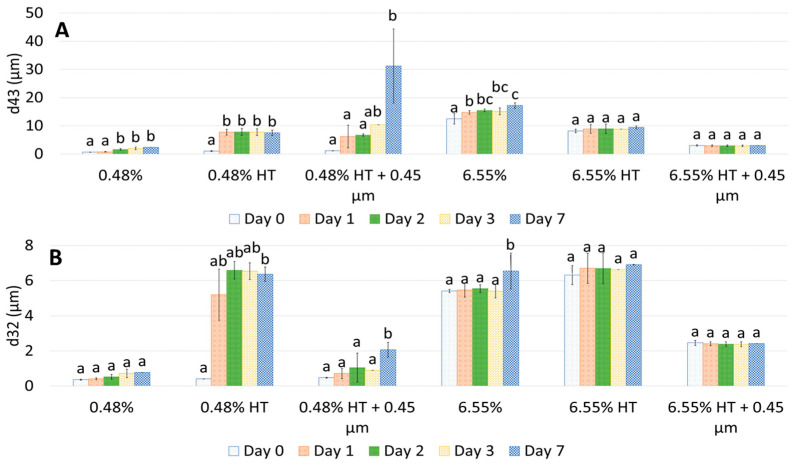
Changes in d_43_ (**A**) and d_32_ (**B**) over 7 days of storage of 10% oil-in-water emulsions made with unmodified, heat-treated (HT), and heat-treated + filtrated (HT + 0.45 μm) GWSC extracts (0.48% and 6.55% wt/wt). A different statistical analysis was applied for each concentration. Samples within the concentration and treatment groups with the same letter(s) did not present significant differences (*p* > 0.05).

**Figure 5:**
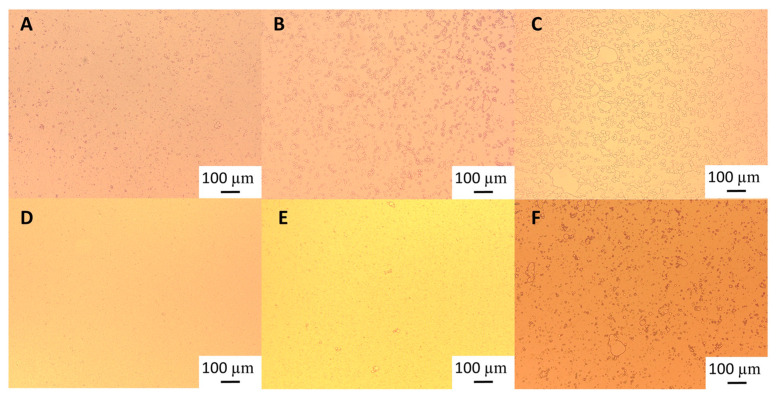
Light microscopy of 10% oil-in-water emulsions of an emulsion stabilised with: 0.48% wt/wt heat-treated (HT) GWSC (**A**) day 0, (**B**) day 1, and (**C**) day 7 and 0.48% wt/wt heat-treated + filtrated (HT + 0.45 μm) GWSC (**D**) day 0, (**E**) day 1, and (**F**) day 7.

Droplet size changes with time in the garlic-based emulsions made with heat-treated extracts, as shown in Figure 4. It seems that the effect of heat treatment of the extracts on emulsion stability also depends on the concentration of the GWSC used. For the lowest concentration (0.48% wt/wt), stability was reduced, and flocculation (Figure 5B) and coalescence (Figure 5C) were observed, occurring more evidently before filtration, whereas for the 0.48% HT + 0.45 µm emulsion, the drastic increase of d_43_ at day 7 seems to be related to droplet aggregation, although coalescence was also observed (Figure 5F). For the higher GWSC concentration (6.55% wt/wt), emulsion stability was improved after heat treatment + filtration. This is likely to be related to the increase in saponin content at the interface of the emulsions made at the highest GWSC after both heat treatment + filtration. Nonetheless, these results are also affected by droplet aggregation (as already shown in Figure 2D–F).

**Figure 6:**
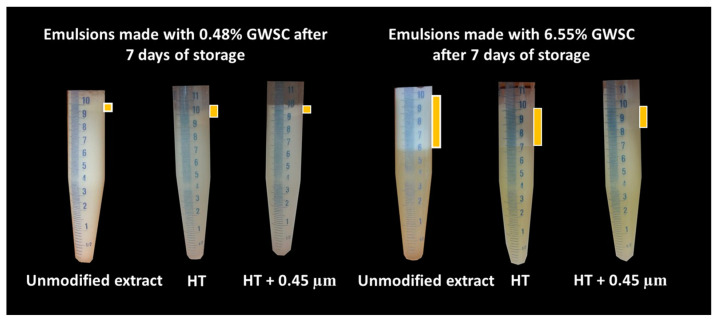
Visual phase separation after 7 days of storage of emulsions made with heat-treated (HT) and heat-treated + filtrated (HT + 0.45 μm) GWSC (0.48 and 6.55% wt/wt).

Visual phase separation of the emulsions after one week of storage can be observed in Figure 6, showing the impact of GWSC concentration and its treatment on creaming stability. As expected, enhanced creaming due to bridging and depletion flocculation, together with the greater droplet size occurring at higher GWSC, was observed, as also reported in our previous research [2]. At both low (0.48% wt/wt) and high (6.55% wt/wt) GWSC, the filtration step in the extracts after heat treatment reduced considerably the phase separation in the emulsions, in agreement with the reduction in the extent of flocculation observed in the micrographs (Figure 5). The underpinning reasons for these results were previously discussed in Section 3.1.1.

### 3.2. Effect of pH on the Emulsification Properties of Garlic Aqueous Extract Compounds

#### 3.2.1. Particle Size Distribution 

**Figure 7:**
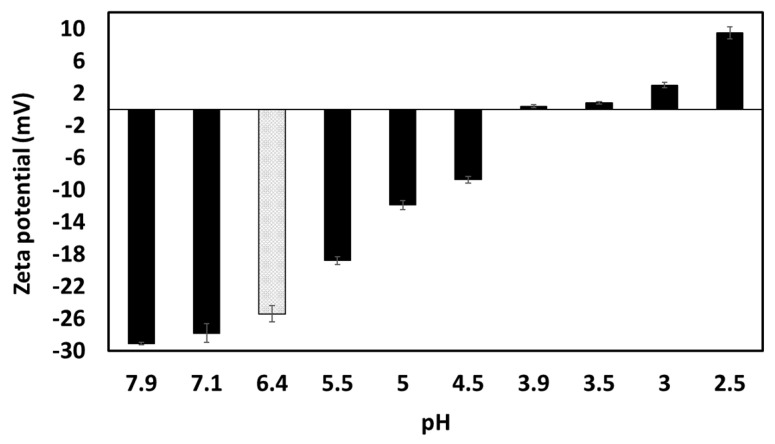
The zeta potential (ζ) of garlic aqueous extract (0.28% GWSC wt/wt concentration) at different pHs. The grey column corresponds to the ζ-potential of the unmodified aqueous extract at native pH.

Unmodified garlic extract had a natural pH of 6.4, and neutral charge was achieved around pH 4 (Figure 7). According to literature, the isoelectric point of garlic proteins is between 4.2–4.5 [27,28] and 5 [29], while saponins remain negatively charged through the whole pH range evaluated here, being more negatively charged at more alkaline pHs [30,31]. The fact that the surface-active compounds found in the extract lost negative charge while changing the pH towards acidic conditions, acquiring a positive zeta potential below pH 4.0, demonstrates that probably the proteins/peptides are dominating the overall charge of the species present in the extract; whereas saponins may lose charge towards low pHs, the proteins start to regain positive charges below their isoelectric point.

**Figure 8:**
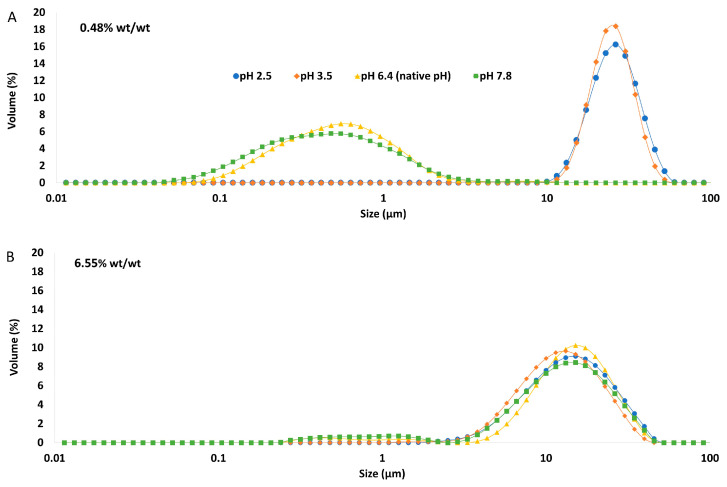
Particle size distribution of 10% oil-in-water emulsions made with pH treated GWSC extracts at (**A**) 0.48% wt/wt and (**B**) 6.55% wt/wt.

**Figure 9:**
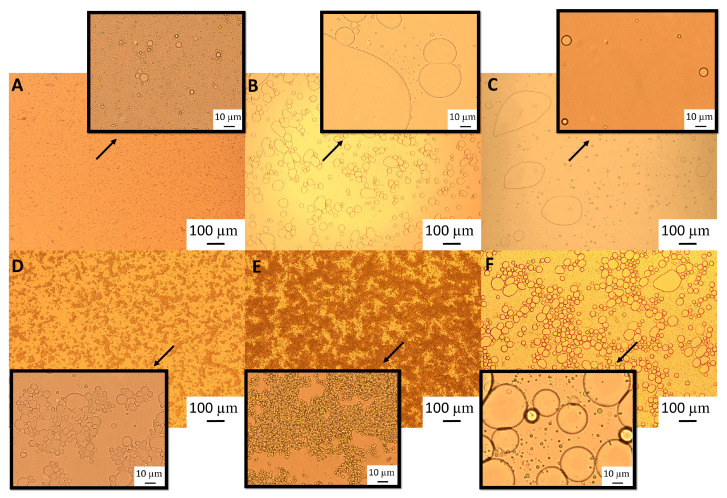
Light microscopy of 10% oil-in-water emulsions made with 0.48% wt/wt GWSC concentration: (**A**) unmodified, (**B**) pH treated (pH 2.5), and (**C**) pH treated + filtrated (pH 2.5 + 0.45 μm), and emulsions made with 6.55% wt/wt GWSC concentration: (**D**) unmodified, (**E**) pH treated (pH 2.5), and (**F**) pH treated + filtrated (pH 2.5 + 0.45 μm).

The particle size distribution of the emulsions comprising the pH-modified extracts (Figure 8) showed that for the lowest GWSC concentration (0.48% wt/wt), a more alkaline pH did not impact the droplet size distribution, while a more acidic pH increased considerably the particle size in the range 10 to 80 µm, as clearly shown in the micrographs of these emulsions (Figure 9B). On the other hand, at the highest GWSC concentration (6.55% wt/wt), emulsions comprising the pH-treated extracts resulted in an overall particle size distribution slightly smaller than the untreated one, with the light microscopy images clearly revealing that when decreasing the pH of the extract, the extent of droplet flocculation is remarkably enhanced, though the actual individual droplets appear smaller (Figure 9E). Since no effect of alkaline pH was observed and the effect of the two tested acid pHs was similar, it was decided to focus only on the pH modification at 2.5, and therefore only extracts with this pH were filtrated and emulsions followed over time. 

**Figure 10:**
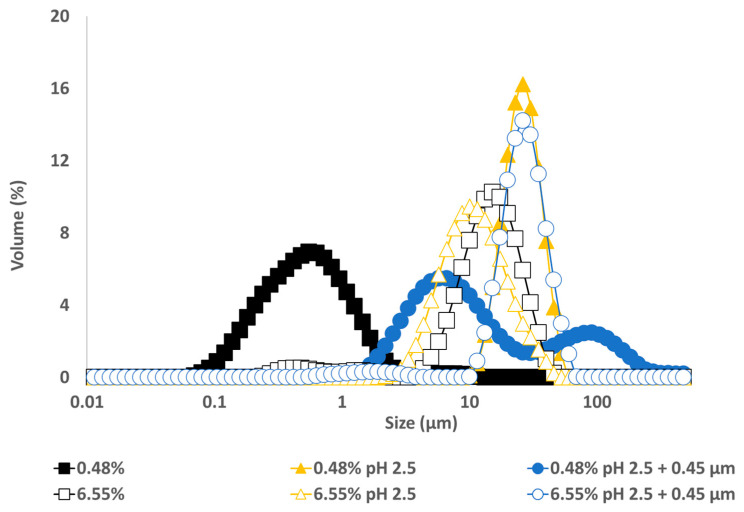
Particle size distribution of 10% oil-in-water emulsions made with unmodified, pH-treated (pH 2.5), and pH-treated + filtrated (pH 2.5 + 0.45 μm) GWSC extracts at two different concentrations.

The particle size distribution of emulsions made with GWSC extracts at pH 2.5, both before and after filtration (Figure 10), showed that the removal of aggregates by filtration at the lowest concentration (0.48% wt/wt) resulted in a bimodal distribution of the droplets. 

This bimodal distribution is composed of a first peak showing smaller droplet sizes than when only changing the pH from 6.4 to 2.5 and a second peak showing bigger droplets in the 100 µm range, in agreement with the micrographs observed for this emulsion (Figure 9C), where also free oil was detected (non-spherical oil visualised on the left side of the mentioned figure). For the highest concentration (6.55% wt/wt), acidification to pH 2.5 resulted in smaller droplet sizes (d_32_ = 8.91 µm), whereas the removal of the aggregates after pH adjustment to 2.5 led to greater droplet sizes (d_32_ = 18.90 µm), as also clearly observed in the micrographs (Figure 9F). It is worthy to note that when measuring droplet size using static light scattering, the samples are diluted in water; therefore, any flocs derived from depletion flocculation are broken; therefore, mainly droplets flocculated via bridging will be detected. Larger individual droplets and clusters of smaller, bridged droplets can generate very similar particle size distributions. This is clearly seen in Figure 9E,F. 

These microscopy images confirmed that changing pH (from 6.4 to 2.5) with or without the removal of large aggregates had a negative effect on the emulsification properties of small concentrations of GWSC. However, very small droplets were observed at high GWSC concentrations (6.55% wt/wt) after only pH modification (from 6.4 to 2.5) (Figure 9E), showing that this could improve the emulsification properties in terms of surface activity, although it came together with a stronger bridging/depletion flocculation phenomenon compared to the unmodified extract (Figure 9D). The only study that assessed the effect of acidification on garlic extracts was that of An et al. [3], although the acidification process was different. These authors exposed their ethanol-extracted GWSC powder to acid hydrolysis at different times. They reported that an increase in the proportion of polysaccharides with a lower molecular weight was correlated with hydrolysis time. They did not observe an effect on droplet sizes, in agreement with our results for the high GWSC concentrations (6.55% wt/wt). The comparison of their results with those of this study should be conducted carefully, as the composition of their garlic-based emulsifier is mainly polysaccharides with some protein traces (the concentration of polysaccharides was ~50 times higher). Although we did not characterise the extracts after their acidification, the initial concentration of polysaccharides in our extract was only ~2–3 times higher than that of proteins.

At pH 2.5, GWSC had a net positive charge (see Figure 7), based on the relative balance between negatively and positively charged components (saponins and proteins, respectively). Böttcher & Drisch [19] reported a decrease in the surface tension of saponin solutions from different sources when decreasing the pH of the media, which makes us believe that the same occurred with the garlic saponins present in our GWSC, adsorbing faster to the interface, although they also reported different foaming stability depending on the saponin’s botanical origin, which shows that not only pH but also molecular structure of saponins is important. Schreiner et al. [24] also reported that acidifying the pH of saponin solutions from different sources was also beneficial for the capacity of the extracts to form emulsions. The improvement of the adsorption of saponins to the interface is probably allowing them to rapidly stabilise oil droplets against re-coalescence, explaining the really small droplets observed in Figure 9E. However, this means that other compounds (proteins and fructans) are more likely to stay in the aqueous phase, thereby potentially contributing to the strong droplet aggregation. This droplet aggregation is probably also related to the lower CMC of saponins at acid pH [23]. 

There is no literature on the effect of pH on the emulsification properties of garlic proteins; however, based on other food proteins, we know that these tend to be more aggregated close to their p*I* on many occasions, leading to greater droplet sizes or even droplet flocculation due to the loss of electrostatic stabilisation [32]. Overall, the fact that these small droplets were not observed with the lowest GWSC compound concentration suggests that the emulsification properties of garlic proteins become worse when decreasing the pH of the aqueous media, and although an acidic pH may improve the emulsification properties of saponins, their concentration at the lowest GWSC content (0.48% wt/wt) is not high enough to stabilise the oil droplets properly, resulting in significantly bigger oil droplets. The fact that after lowering the pH and removing the big aggregates, 6.55% wt/wt GWSC extract resulted in bigger droplet sizes than after only changing the pH suggests that saponin micelles were removed after filtration. However, to better understand the underpinning reasons, separating the different biopolymers/surfactants and testing their emulsification capacity individually would be needed.

#### 3.2.2. Emulsion Stability over Time

**Figure 11:**
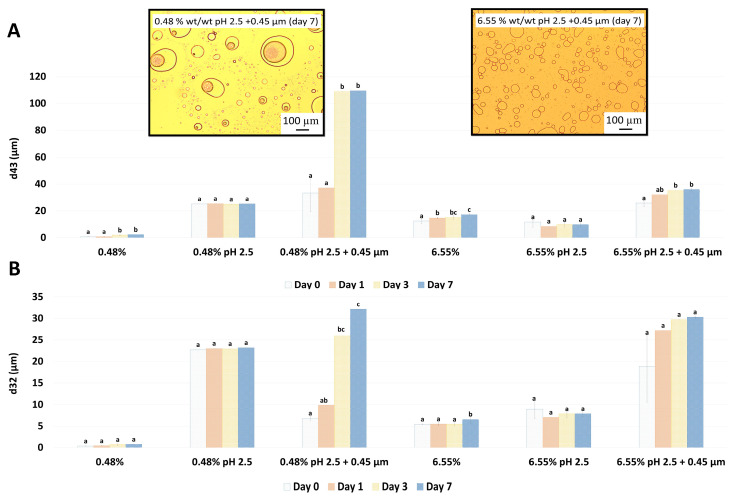
Changes in d_43_ (**A**) and d_32_ (**B**) over 7 days of storage of 10% oil-in-water emulsions made with unmodified, pH-treated (pH 2.5), and pH-treated + filtrated (pH 2.5 + 0.45 μm) GWSC at two different concentrations. A different statistical analysis was applied for each concentration. Samples within the concentration and treatment groups with the same letter(s) did not present significant differences (*p* > 0.05).

Droplet size changes with time for the garlic-based emulsions made with pH treated extracts (pH 2.5) are shown in Figure 11. When adjusting only pH to 2.5 for both GWSC concentrations, the emulsions did not change droplet size over time. These results are in disagreement with those in An et al. [3], who observed an increase in droplet size over time in their emulsions stabilised with garlic extracts. Differences can be related to the different acidification methods, different protein-polysaccharide ratios, and percentages of oil (1% versus 10% wt/wt), which all can result in different interfacial compositions. However, storage had a strong negative effect on the droplet size of emulsions made with the acidified GWSC extracts after the removal of larger aggregates, and coalescence occurred, more evidently at the lowest GWSC concentration. This is likely due to the decrease in biopolymers at the interface, which protects emulsions against close contact between coated droplets via steric stabilisation [4]. In agreement with this, An et al. [3] reported that the destruction of the chain conformation of the branched polysaccharides by acid hydrolysis resulted in a thin interfacial layer during emulsification and limited steric repulsion, insufficient to prevent flocculation/coalescence phenomena [33].

**Figure 12:**
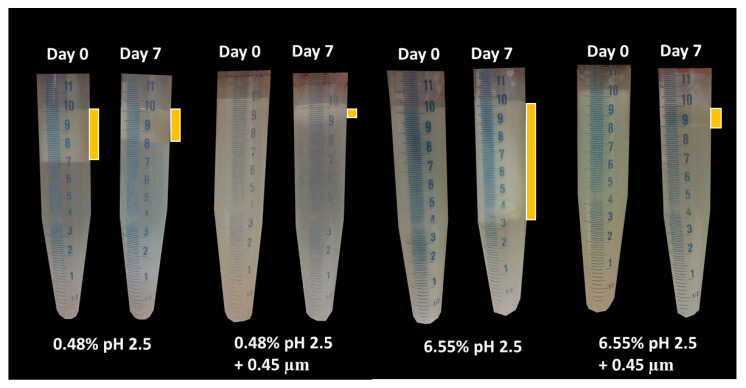
Visual phase separation over time of emulsions made with pH-treated (pH 2.5) and pH-treated + filtrated (pH 2.5 + 0.45 μm) GWSC extracts (0.48 and 6.55% wt/wt).

The visual phase separation of these emulsions can be observed in Figure 12. For the lowest GWSC concentration (0.48% wt/wt), strong phase separation was observed after pH modification of the extract (from 6.4 to 2.5). Interestingly, this strong phase separation was not observed after both pH modification and the removal of aggregates from the extract. This is probably affected by both the presence of free oil and the removal of the big aggregates producing droplet aggregation. For the highest GWSC concentration (6.55% wt/wt), phase separation after pH modification or after both pH modification and removal of aggregates from the extract did not occur at day 0; however, was observed after 7 days of storage. On day 7, the same trend as with the lower GWSC concentration was observed, probably having the same underpinning reasons.

## 4. Conclusions

The emulsification properties of GWSC (encompassing garlic proteins, saponins, and carbohydrates) are affected by GWSC concentration, heat treatment, and acidification. When compared to untreated extracts it can be concluded that: (i) Heat treatment and pH modification of GWSC followed by filtration at low concentrations results in emulsions with bigger droplet sizes, highly likely related to their low saponin concentration, not able to overcome the reduction of the emulsifying capacity of the other surface-active compounds (proteins and peptides) after heat treatment/acidification; and (ii) Heat treatment and pH modification of GWSC at high concentrations results in emulsions with smaller droplet sizes but stronger flocculation that for the heat treated extracts could be reduced after the removal of biopolymer aggregates by filtration (filter pores 0.45 µm). The same cannot be said for the emulsions made with acidified GWSC, as the removal of these aggregates resulted in a large reduction of surface-active compounds, which led to bigger droplets in the final emulsion.

The underpinning reasons for these results are related to the coexistence of different surface-active compounds (proteins, saponins, and carbohydrates) and their interactions after GWSC modifications, which limits the extrapolation/comparison of the results.

This study constitutes an important contribution to the development of healthy and sustainable food products (e.g., beverages or sauces). Further research should be conducted regarding quantification, molecular characterization, and separation of the individual GWSC, as well as the notability of garlic saponins and proteins, as this will help to better elucidate the mechanisms behind the observations presented in this study. 

## Figures and Tables

**Table 1:** Approximate composition of various garlic aqueous extracts *.

GWSC (%)	Water (%)	Proteins (%)	Carbohydrates (%)	Saponins (%)	Other Components (%)
0.48	99.52	0.1 ± 0.01	0.21 ± 0.03	0.06 ± 0.01	No data
6.55	93.45	1.06	3.45 ± 0.75	0.9 ± 0.12	1.14

* Data extracted from Bravo-Núñez et al. [2] and current analysis with the permission of the authors.

## Data Availability

Data are available upon request from the corresponding author.
